# A Randomized Study of Myostaal® Liniment as an Add-On Therapy for Muscle Strengthening in Cases of Knee Osteoarthritis

**DOI:** 10.7759/cureus.68012

**Published:** 2024-08-28

**Authors:** Shailesh Deshpande, Vaishali Deshpande, Noopur Bhatt, Bhavin Dhanavade, Hemant Toshikane, Bhagawan G Kulkarni, Mukesh Chawda, Megha Nalawade, Rajmohan Seetharaman

**Affiliations:** 1 Department of Internal Medicine, Parul Institute of Ayurved, Parul University, Vadodara, IND; 2 Department of Internal Medicine, Parul Institute of Ayurved and Research, Parul University, Vadodara, IND; 3 Department of Physiotherapy, Parul Institute of Physiotherapy, Parul University, Vadodara, IND; 4 Department of Pharmacology and Therapeutics, Parul Institute of Ayurved and Research, Parul University, Vadodara, IND; 5 Department of Surgery, Parul Institute of Ayurved, Parul University, Vadodara, IND; 6 Department of Anatomy, Parul Institute of Ayurved and Research, Parul University, Vadodara, IND; 7 Department of Medical Services, Solumiks Herbaceuticals Limited, Mumbai, IND; 8 Department of Clinical Research, Shree Dhootapapeshwar Limited, Mumbai, IND; 9 Department of Pharmacology, MGM Medical College and Hospital, MGM Institute of Health Sciences, Navi Mumbai, IND

**Keywords:** degenerative musculoskeletal conditions, joint functionality, visual analogue scale score, six-minute walk test, knee muscle strength total womac score

## Abstract

Introduction: Knee osteoarthritis (OA) is a prevalent degenerative musculoskeletal condition, affecting approximately 277 million people worldwide, with significant impacts on mobility, especially in women and obese patients, and an increasing incidence among Indians aged 30 to 50 years. The primary objective was to evaluate the knee muscle-strengthening effect of Myostaal® liniment (Solumiks Herbaceuticals Limited, Mumbai, India) as an add-on to physiotherapy for 90 days compared to physiotherapy alone in participants with knee OA. Secondary objectives included assessing changes in the total Western Ontario and McMaster Universities Osteoarthritis Index (WOMAC) score, WOMAC Subscale scores, Six-Minute Walk Test (6MWT) distance, Single Leg Stance Test (SLST) duration, Visual Analogue Scale (VAS) score, and the number of adverse events from baseline to Day 90 between the two groups.

Methods: Seventy participants were randomly allocated to Group A (Myostaal® liniment plus physiotherapy) or Group B (physiotherapy alone) for 90 days, with Myostaal® liniment applied twice daily in Group A. Data were recorded in Case Report Forms (CRFs) and analyzed using parametric tests for within-group comparisons (one-way ANOVA or Friedman test) and non-parametric tests (Mann-Whitney test) for between-group comparisons, with significance set at p<0.05.

Results: The knee muscle strength (index knee) in Group A (test medication group) was significantly greater compared to Group B (standard treatment group) at Visit 3 (p<0.05; Day 60±3) and Visit 4 (p<0.001; Day 90±3). For the non-index (other) knee, a statistically significant increase in knee muscle strength was observed (p<0.001 at Day 90±3) solely in Group A. A notable reduction in total WOMAC score was seen in Group A from Visit 2 (p<0.01; Day 30±3) onward, compared to Visit 1 (Day 0). The scores at Visit 3 (p<0.001; Day 60±3) and Visit 4 (p<0.001; Day 90±3) were significantly lower than those at Visit 2 (Day 30±3).

Conclusion: The local application of Myostaal® liniment through massage as an adjunct to a physiotherapy regimen, improved knee muscle strength in participants with knee OA, leading to an enhancement in joint functionality. Additionally, Myostaal® liniment provided superior pain relief as an add-on therapy.

## Introduction

Knee osteoarthritis (OA) is a common degenerative musculoskeletal condition encountered in clinical practice, and its prevalence is expected to increase as the population ages. The estimates indicate that approximately 277 million (3.8%) people globally are affected by knee OA. Knee OA is estimated to affect 3.9% of rural Indians and 5.5% of urban Indians [1]. Osteoarthritis continues to have a serious impact on the lives of elderly people, but in the last few decades, Indians in the age group of 30 to 50 years are falling prey to this disease. By 2025, India is estimated to be the chronic disease capital, with 60 million people suffering from arthritis. Knee OA involves progressive loss of articular cartilage and bone remodeling, often starting in the third or fourth decade, and is more common in women and obese patients. It leads to significant mobility impairment, especially in females. Risk factors include excess weight, joint trauma, developmental deformities, quadriceps muscle weakness, and abnormal tibial rotation. Knee OA has a slow, progressive onset, with pain increasing with activity and reducing with rest. It is classified as primary when due to aging or overuse and secondary when resulting from causes like trauma or infection. The condition arises from an imbalance between mechanical stress on the joint and tissue resilience, linked to decreased muscle strength and altered joint biomechanics [2]. Hence, treatment of early knee OA may be very effective if conscientiously carried out.

To prevent the surgery and preserve joint mobility, knee strengthening exercises are an important aspect of treatment. Exercises (e.g., muscle strengthening and aerobic exercises) have been proven to be effective in pain management and in improving physical functioning [2]. Conventional treatments for OA include pain medication (nonsteroidal anti-inflammatory drugs (NSAIDs) and cyclooxygenase-2 inhibitors), exercises, hot and cold therapy, corticosteroid injections, and eventually, surgery to repair the joint [3]. Despite conventional treatment, OA is often progressive and frequently leads to chronic pain and disability [3]. Knee OA has a significant negative impact on health-related quality of life [4]. Quadriceps strengthening makes a surprising difference in improving pain, function, and quality of life of patients with knee OA [5]. Strong quadriceps can delay the necessity for surgery and dependency on medicines. Since bone density is affected by OA, resistance training and weight-bearing exercises are the best ways to improve bone density and muscle function. While standard methods such as exercises and pain medications are effective, they may not fully alleviate symptoms or prevent disease progression. Incorporating effective topicals into physiotherapy for knee OA can enhance treatment outcomes by addressing both symptomatic relief and supportive muscle strengthening.

Ayurveda provides a rich array of ingredients and formulations for managing musculoskeletal disorders like OA, addressing both the root cause and offering symptomatic relief. Ayurveda practitioners often recommend massages with medicated oils such as mahanarayan oil (tel) and nirgundi (*Vitex negundo*) tel to enhance the tone of peri-articular muscles. Myostaal® liniment (Solumiks Herbaceuticals Limited, Mumbai, India), a proprietary Ayurvedic preparation available in the Indian market for over 30 years, is extensively used for managing knee OA and other musculoskeletal conditions. It incorporates mahanarayan tel to improve muscle tone and maintain joint alignment, as well as nirgundi tel, gandapura (*Gaultheria fragrantissima*), eucalyptus (*Eucalyptus globulus*) tel, devadaru (*Cedrus deodara*), and sarala (*Pinus longifolia*) to alleviate joint pain, swelling, and morning stiffness [6-11]. Additionally, Myostaal® liniment contains sesame oil (tila, *Sesamum indicum*), known for its benefits in improving muscle tone and strength, and recent studies show it has an analgesic effect comparable to Diclofenac gel for reducing knee OA pain [12].

The primary objective of the study was to evaluate the knee muscle strengthening effect of Myostaal® liniment application as an add-on to physiotherapy for 90 days, compared to physiotherapy alone, in participants suffering from knee OA. Secondary objectives included assessing changes in the total Western Ontario and McMaster Universities Osteoarthritis Index (WOMAC) score and WOMAC Subscale scores from baseline to Day 90, changes in the distance covered in the Six-Minute Walk Test (6MWT) from baseline to Day 90, changes in Single Leg Stance Test (SLST) duration from baseline to Day 90, and changes in Visual Analogue Scale (VAS) score from baseline to Day 90, all with Myostaal® liniment application as an add-on to physiotherapy compared to physiotherapy alone. The secondary objectives were designed to provide insights into how the interventions might affect pain relief, functional capacity, and overall quality of life. Additionally, the study aimed to compare the number of participants showing adverse events from baseline to Day 90 between the Myostaal® liniment plus physiotherapy group and the physiotherapy alone group.

## Materials and methods

Study approvals

This prospective, randomized, active-controlled, two-arm, parallel-group study was conducted at two sites within Parul University, Vadodara, India. The study was initiated after receiving ethics committee approval from the Khemdas Ayurveda Hospital, Parul Institute of Ayurved and Research (Reference: PIAR/IEC/691/2020, Date: September 11, 2020), and Parul Institute of Ayurved (Reference: PU/PIA/IECHR/2020/234, Date: August 5, 2020). Following ethics approval, the study received approval from the Clinical Trials Registry-India (CTRI) on November 10, 2020 (CTRI registration No.: CTRI/2020/11/029031). Before the commencement of the trial, written informed consent was obtained from all participants, ensuring that they were fully aware of the study's objectives and procedures.

Subject selection criteria

Participants aged 40 to 70, of either gender, diagnosed with unilateral idiopathic knee OA for one month to five years according to American College of Rheumatology guidelines, were included in the study through convenience sampling. Eligible individuals had to exhibit at least three of the following: age over 38 years, morning stiffness within 30 minutes of walking, crepitus, bony tenderness, bony enlargement, and no palpable warmth. Inclusion required no prior or only conservative treatment, baseline knee joint pain over 40 mm on the VAS in the past 24 hours, and radiological findings of Grades 1 and 2 per the Kellgren and Lawrence system. Participants had to provide written informed consent and attend follow-up visits.

Exclusion criteria included secondary arthritis, systemic inflammatory arthritis, recent NSAIDs or corticosteroid use, knee surgery history or upcoming surgery within three months, and knee replacement surgery or arthroscopy in the past two years. Also excluded were individuals with recent intra-articular viscosupplementation, ongoing use of muscle relaxants or antidepressants for chronic pain, autoimmune disease, uncontrolled hypertension, uncontrolled diabetes mellitus, chronic severe respiratory disease, renal, hepatic, or peptic ulcer disease. Further exclusions comprised a history of life-threatening cardiovascular or neurological events, alcohol or drug abuse, bleeding disorders, severe active infectious diseases, severe allergies or anaphylactic reactions, HIV, hepatitis B or C infections, pregnancy, lactation, recent participation in another clinical trial, mental retardation, psychiatric illness, or unconsciousness.

Study interventions

Participants were screened for knee OA at the internal medicine (kayachikitsa) OPD of both study sites, and those meeting the inclusion criteria were invited to participate. Written informed consent was obtained from all eligible participants before any study procedures were conducted. Following confirmation of a radiological diagnosis of unilateral knee OA, participants were randomly allocated (1:1) into one of two groups: Group A (test medication group) or Group B (standard treatment group). Test medication consisted of Myostaal® liniment, which is used for symptomatic relief and improved muscle tone in conditions such as knee osteoarthritis, lumbago, sciatica, cervical and lumbar spondylosis, tenosynovitis, and low back pain during pregnancy.

Participants in Group A were instructed to apply 5 ml of Myostaal® liniment to both knees twice daily and follow a physiotherapy regimen for 90 days. They were advised to avoid application on open wounds, mucous membranes, and inflamed or tender skin and to discontinue use if irritation or an allergic reaction occurred. Each participant received a total of 660 ml of Myostaal® liniment, distributed as six 110-ml bottles, during each of the three visits (Visit 1, Visit 2, and Visit 3), sufficient for approximately 30±3 days of use. In contrast, participants in Group B followed only the physiotherapy regimen for 90 days. Myostaal® liniment application protocol involved applying 5 ml twice daily, once in the morning and once in the evening, for eight weeks. Participants were instructed to rub the liniment gently into the targeted area in circular motions until fully absorbed, avoiding broken or irritated skin. They were also advised to wash their hands thoroughly after application and avoid contact with eyes and mucous membranes.

To assess treatment effectiveness, muscle strength was measured with a handheld dynamometer at baseline and the end of the treatment period. The WOMAC Osteoarthritis Index evaluated pain, stiffness, and physical function at both the start and conclusion of the study. The 6MWT was performed in a standardized 30-meter corridor, with participants walking at their own pace for six minutes. All assessments were conducted at the same times of day to ensure consistency and minimize variability. During the initial visit (Day 0), a physiotherapist demonstrated and instructed participants on the physiotherapy exercises, which were to be performed twice daily with five repetitions of each exercise. The knee OA regimen included a structured program designed to improve joint function and alleviate pain through strength training, flexibility exercises, and aerobic conditioning. Sessions were typically held two to three times per week over eight to 12 weeks, with the exercises and frequency adjusted based on each patient's individual needs and clinical condition. A detailed visit-wise schedule is given in Table 1.

**Table 1:** Visit-wise schedule of the study participants WOMAC: Western Ontario and McMaster Universities Arthritis Index

No.	Assessment	Baseline visit	Visit 1	Visit 2	Visit 3	Visit 4
		Day 3 to Day 7	Day 0	Day 30±3	Day 60±3	Day 90±3
1	Informed consent	✓	-	-	-	-
2	History and physical examination (vital parameters, systemic examination, and BMI)	✓	✓	✓	✓	✓
3	X-Ray knee, anteroposterior (AP)-lateral	✓	-	-	-	-
4	Blood investigations					
	Complete blood count (CBC)	✓	-	-	-	-
	Random blood sugar (RBS)	✓	-	-	-	-
5	Randomization	-	✓	-	-	-
6	Medication dispensing	-	✓	✓	✓	-
7	Medication compliance	-	-	✓	✓	✓
8	Knee muscle strength assessment with dynamometer	-	✓	✓	✓	✓
9	Total WOMAC score WOMAC subscale scores	-	✓	✓	✓	✓
12	Six-Minute Walk Test	-	✓	✓	✓	✓
13	Single Leg Stance Test	-	✓	✓	✓	✓
14	Pain assessment with Visual Analogue Scale (VAS)	✓	✓	✓	✓	✓
15	Adverse events	-	✓	✓	✓	✓

Data analysis

The total duration of the study was 11 months, from 1 July 2021 to 31 May 2022, which included the time needed for data analysis and report writing. The first participant was recruited on 1 July 2021, and the final visit for the last participant occurred on 27 January 2022. The targeted sample size of 60 participants, with 30 allocated to each group, was selected to align with the pilot exploratory nature of the study. A retrospective power analysis was subsequently performed to ensure that the study was sufficiently powered.

All data were meticulously recorded in the Case Report Forms (CRFs) by the study investigator. Upon completion of the study, parametric data were presented as mean±SD, while non-parametric data were shown as median (range). For within-group comparisons, one-way ANOVA or the Friedman test was utilized, and the Mann-Whitney test was applied for between-group comparisons. A significance level of p<0.05 was considered for all statistical analyses.

## Results

Baseline variables of participants

**Figure 1:**
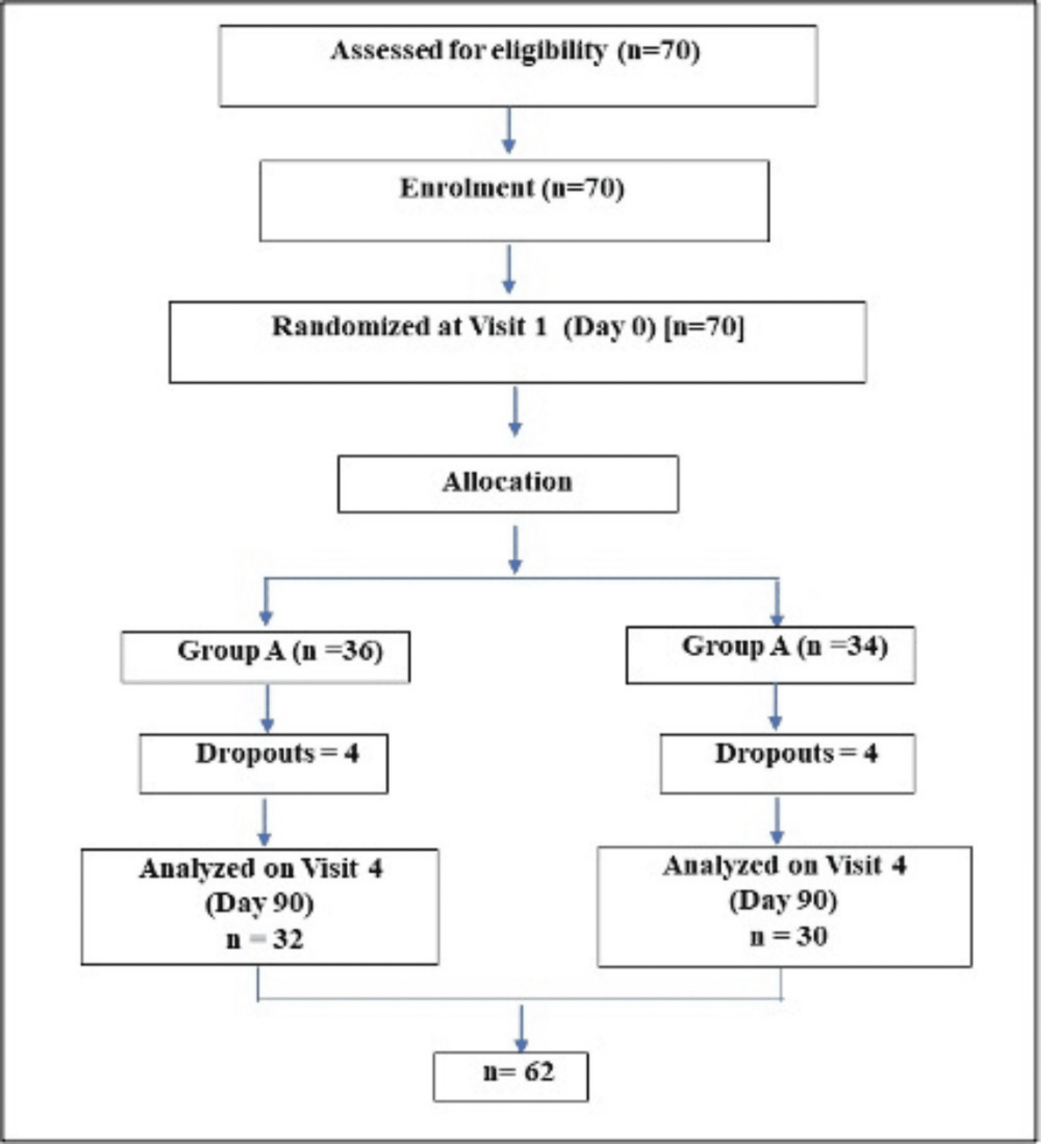
A CONSORT flow diagram of the study and the participants CONSORT: Consolidated Standards of Reporting Trials

Seventy participants suffering from unilateral knee OA were recruited in the study. There were no screen failures. Out of the 70 participants, 36 participants were recruited in Group A, and 34 participants were recruited in Group B based on the randomization procedures. Sixty-two participants completed the study: 32 participants in Group A and 30 participants in Group B. In total, there were eight dropouts, four each in Group A and Group B. The reason for all dropouts in Group A was the migration of participants to different locations. In Group B, two participants migrated to different locations, one participant lost to follow-up for unknown reasons, and one participant withdrew the consent due to inadequate relief (Figure 1).

affNo significant difference was observed in the mean age and body mass index (BMI) of participants in both groups. The other baseline variables, like complete blood count (CBC) and random blood sugar (RBS), were comparable between both groups (Table 2). At baseline, it was observed that unilateral knee OA affected 41 (66%) participants in the age group of 60-70 years and 39 (61%) females (Table 3).

**Table 2:** Baseline variables of the participants Values are expressed as mean±SD.

Variables	Group A (n=32) (Mean±SD)	Group B (n=30) (Mean±SD)
Age (years)	60.92±8.28	58.41±6.70
BMI (kg/m^2^)	24.059±3.98	25.72±5.75
Platelets (cells/mm^3^)	274688.46±75328.79	271642.86±49606.46
Red blood cells (million/mm^3^)	4.17±0.61	4.30±0.58
Total leucocyte count (cells/mm^3^)	7460.77±1124.83	7685.71±2088.70
Random blood sugar (RBS) (mg/dL)	97.92±20.58	103.75±24.60

**Table 3:** Subgroup analysis of age and gender distribution Group A (test medication group): Myostaal® liniment massage followed by a physiotherapy regimen for 90 days; Group B (standard treatment group): physiotherapy regimen for 90 days

Variables		Group A (n=32)	Group B (n=30)
Age (years)	40-49 years	2 (6.2%)	4 (13.33%)
	50-59 years	7 (21.9%)	8 (26.66%)
	60-70 years	23 (71.9%)	18 (60%)
Gender	Male	10 (31.25%)	13 (43.33%)
	Female	22 (68.75%)	17 (56.66%)

Primary efficacy parameters

Comparison of Knee Muscle Strength in Group A and Group B

**Figure 2:**
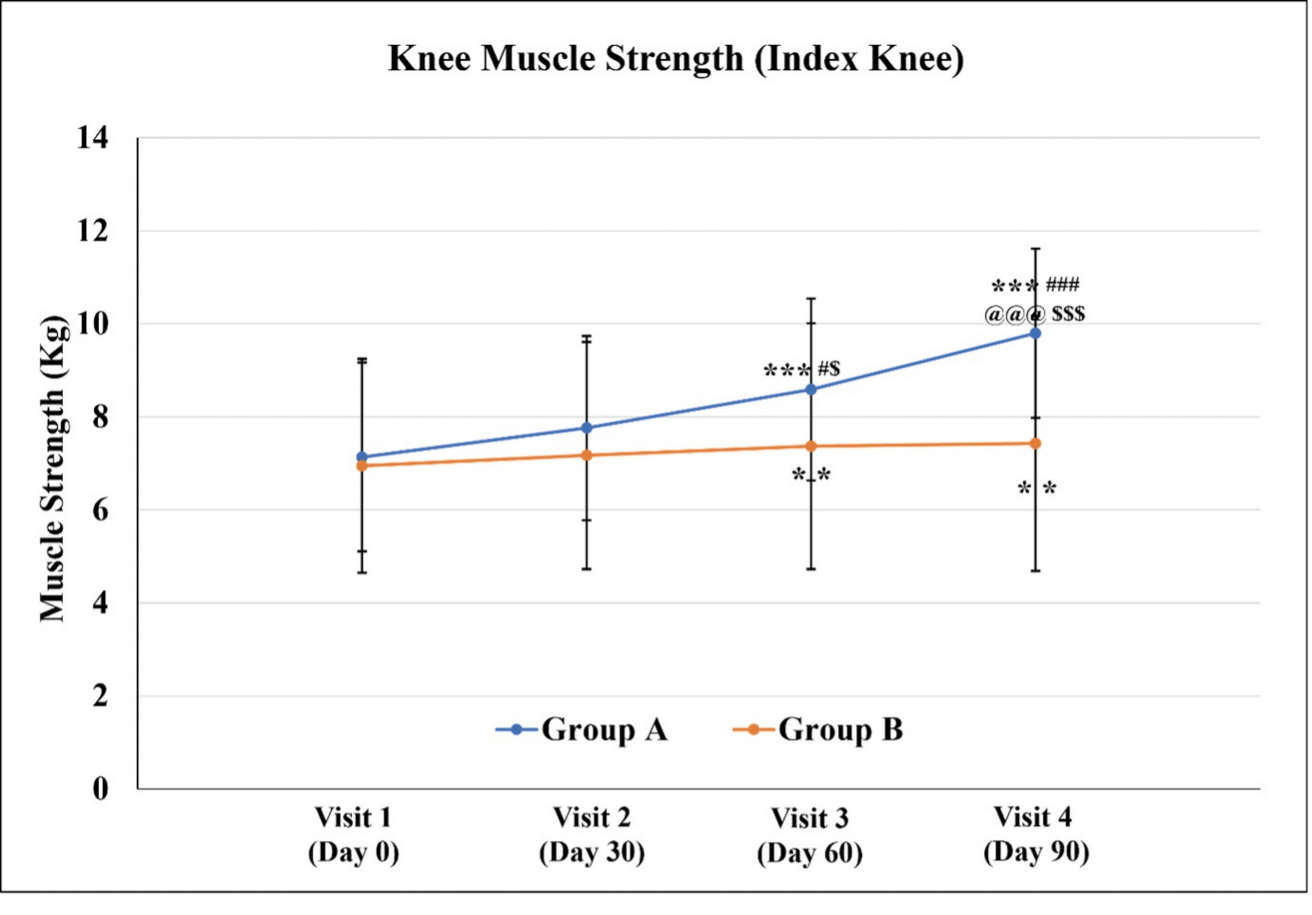
Knee muscle strength (index knee) Values are expressed as Mean±SD. ^**^p<0.01 and ^***^p<0.001 as compared to Visit 1; ^#^p<0.05 and  ^###^p<0.001 as compared to Visit 2; and ^@@@^p<0.001 as compared to Visit 3 using repeated measure ANOVA; ^$^p<0.05 and ^$$$^p<0.001 as compared to Group B using an unpaired t-test.

For the index (affected) knee, a significant increase in knee muscle strength was seen in both the groups at Visit 3 (Day 60±3) and Visit 4 (Day 90±3) as compared to the respective readings on Visit 1 (Day 0). Only Group A exhibited a significant increase in knee muscle strength on Visit 3 and Visit 4 as compared to Visit 2 (Day 30±3) and Visit 3 (Day 60±3) respectively. The knee muscle strength in Group A was significantly higher as compared to Group B on Visit 3 and Visit 4 (Figure 2 and Table 4).

**Table 4:** Knee muscle strength (index knee) Values are expressed as Mean±SD. ^**^p<0.01 and ^***^p<0.001 as compared to Visit 1; ^#^p<0.05 and ^###^p<0.001 as compared to Visit 2; ^@@@^p<0.001 as compared to Visit 3 using repeated measure ANOVA; ^$^p<0.05 and ^$$$^p<0.001 as compared to Group B using an unpaired t-test.

Visit	Index (affected) knee	
	Group A (n=32)	Group B (n=30)
Visit 1 (Day 0)	7.14 ± 2.30	6.95 ± 2.03
Visit 2 (Day 30 ± 3)	7.76 ± 2.44	7.17 ± 1.98
Visit 3 (Day 60 ± 3)	8.59 ± 2.64^***#$^	7.37 ± 1.96**
Visit 4 (Day 90 ± 3)	9.80 ± 2.74***^###@@@$$$^	7.43 ± 1.82**

**Figure 3:**
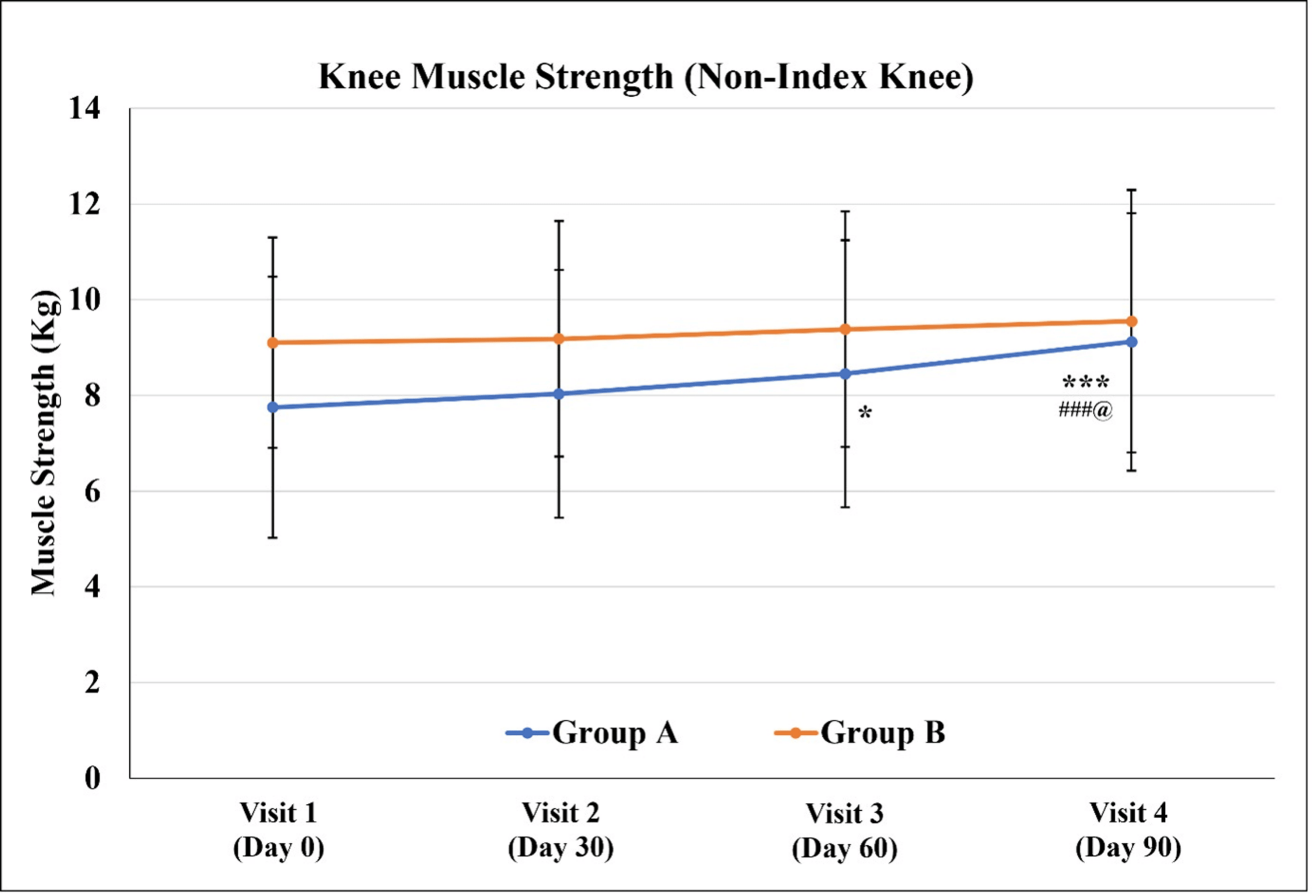
Knee muscle strength (non-index knee) Values are expressed as Mean ± SD. ^*^p<0.05 and ^***^p<0.001 as compared to Visit 1; ^###^p<0.001 as compared to Visit 2; ^@^p<0.05 as compared to Visit 3 using repeated measure ANOVA.

For the non-index (other) knee, both groups exhibited an increase in knee muscle strength, but it was statistically significant only in Group A. Although the knee muscle strength in Group A was significantly lower as compared to Group B at Visit 1, no such difference was noted on Visit 3, indicating better efficacy in Group A (Figure 3 and Table 5).

**Table 5:** Knee muscle strength (non-index knee) Values are expressed as Mean ± SD. ^*^p<0.05, ^**^p<0.01 and ^***^p<0.001 as compared to Visit 1; ^#^p<0.05 and ^###^p<0.001 as compared to Visit 2; ^@^p<0.05 and ^@@@^p<0.001 as compared to Visit 3 (Day 60±3) using repeated measure ANOVA; ^$^p<0.05 and ^$$$^p<0.001 as compared to Group B using an unpaired t-test.

Visit	Non-index (other) Knee	
	Group A (n=32)	Group B (n=30)
Visit 1 (Day 0)	7.75 ± 2.73^$^	9.1 ± 2.20
Visit 2 (Day 30 ± 3)	8.03 ± 2.59	9.18 ± 2.46
Visit 3 (Day 60 ± 3)	8.45 ± 2.79*	9.38 ± 2.46
Visit 4 (Day 90 ± 3)	9.12 ±2.69***^###@^	9.55 ± 2.74

Secondary efficacy parameters

*Comparison of the Total WOMAC Score and *WOMAC *Subscale Scores in Group A and Group B*

**Figure 4:**
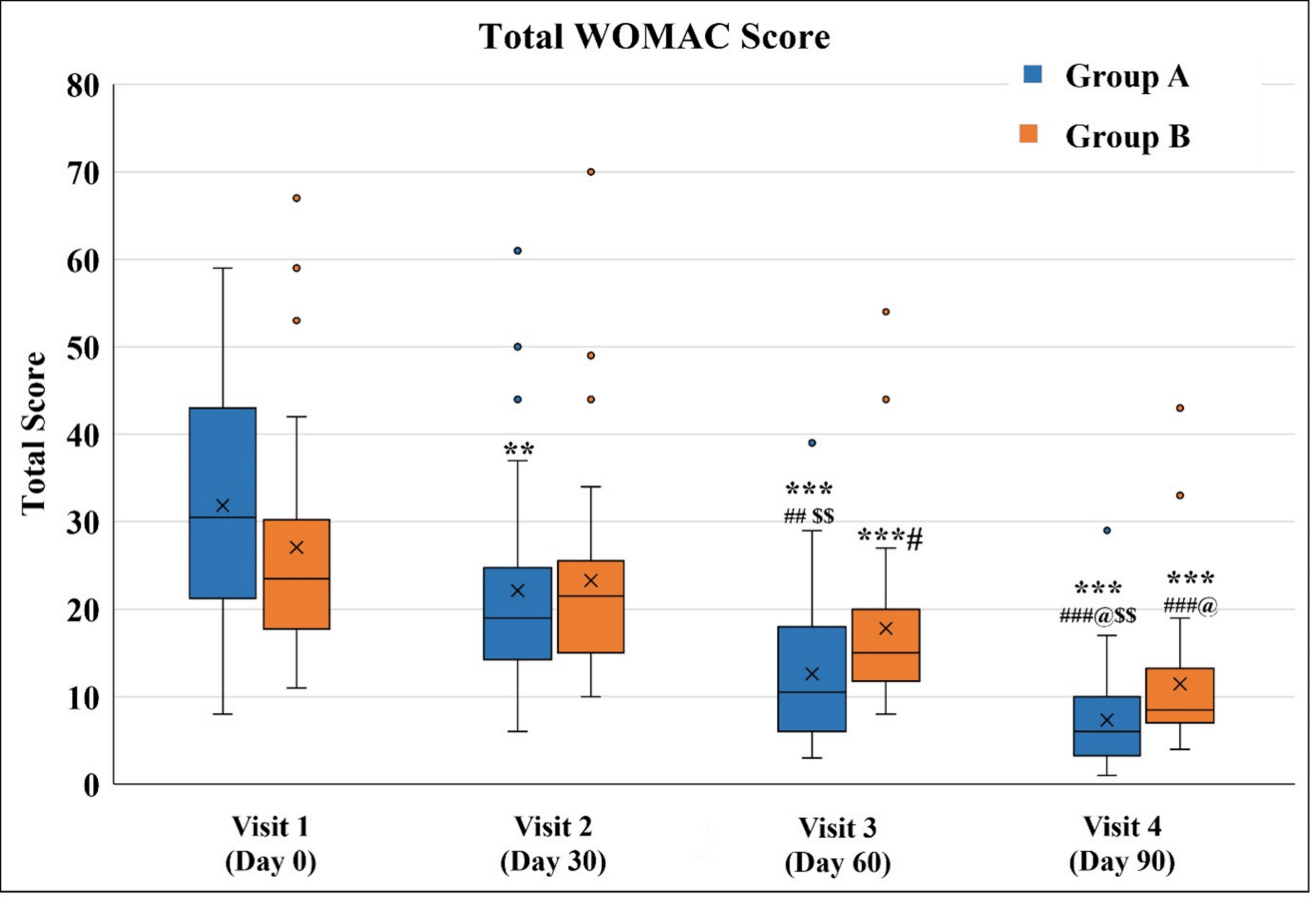
Total WOMAC Score ^**^p<0.01 and ^***^p<0.001 as compared to Visit 1; ^#^p<0.05, ^##^p<0.01, and ^###^p<0.001 as compared to Visit 2; ^@^p<0.05 as compared to Visit 3 using the Friedman test; ^$$^p<0.01 as compared to Group B using the Mann-Whitney test. WOMAC: Western Ontario and McMaster Universities Osteoarthritis Index

A significant decrease in Total WOMAC Score was observed in Group A from Visit 2 onwards as compared to Visit 1. The score on Visit 3 and Visit 4 was significantly lower even as compared to the score on Visit 2. Also, the score on Visit 4 was significantly lower as compared to the score on Visit 3). Although Group B also exhibited a decrease in the total WOMAC score, it was significant only after Visit 3. Group A exhibited a significantly lower score as compared to Group B on Visit 3 and Visit 4, indicating better symptomatic relief (Figure 4 and Table 6).

**Table 6:** Total WOMAC Score ^**^p<0.01 and ^***^p<0.001 as compared to Visit 1; ^#^p<0.05, ^##^p<0.01, and ^###^p<0.001 as compared to Visit 2; ^@^p<0.05 as compared to Visit 3 using the Friedman test; ^$^p<0.05, ^$$$^p<0.001 as compared to Group B using the Mann-Whitney test. WOMAC: Western Ontario and McMaster Universities Osteoarthritis Index

Visit	Group A (n=32)	Group B (n=30)
Visit 1 (Day 0)	30.5 (8-59)	23.5 (11-67)
Visit 2 (Day 30 ± 3)	19 (6-50)**	21.5 (10-70)
Visit 3 (Day 60 ± 3)	10.5 (3-39) ***^##$$^	15 (8-54) ***^#^
Visit 4 (Day 90 ± 3)	6 (1-29) ***^###@$$^	8.5 (4-43) ***^###@^

**Figure 5:**
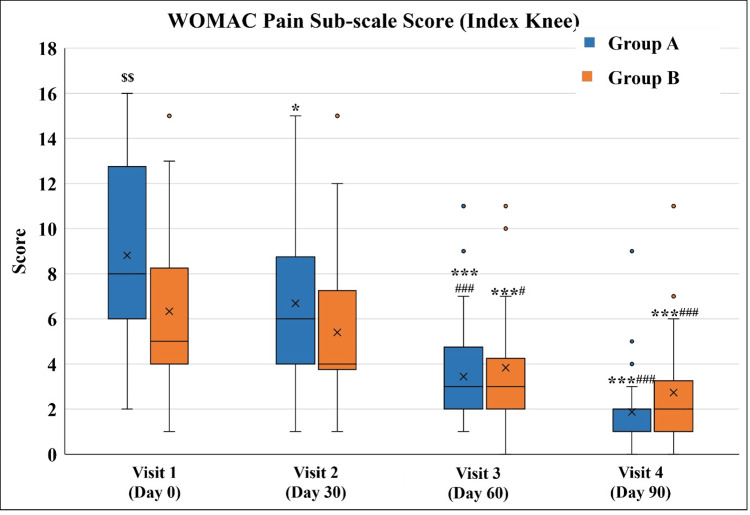
WOMAC Pain Subscale score ^*^p<0.05 and ^***^p<0.001 as compared to Visit 1; ^#^p<0.05 and ^###^p<0.001 as compared to Visit 2 using the Friedman test; ^$$^p<0.01 as compared to Group B using the Mann-Whitney test. WOMAC: Western Ontario and McMaster Universities Osteoarthritis Index

A gradual decrease in the WOMAC Pain Subscale score was observed in both groups. It was significant in Group A from Visit 2 onwards, whereas in Group B it was significant from Visit 3. In both groups, the scores on Visit 3 and Visit 4 were significantly lower than on Visit 2. Interestingly, the scores in both groups were significantly different at Visit 1 (median pain score of eight in Group A vs. five in Group B), while no such difference was noted on Visit 4 indicating better pain reduction in Group A (Figure 5 and Table 7).

**Table 7:** WOMAC Pain Subscale score ^*^p<0.05 and ^**^p<0.01, ^***^p<0.001 as compared to Visit 1; ^#^p<0.05 and ^###^p<0.001 as compared to Visit 2 using the Friedman test; ^$$^p<0.01 as compared to Group B using the Mann-Whitney test. WOMAC: Western Ontario and McMaster Universities Osteoarthritis Index

Visit	WOMAC pain Subscale score	
	Group A (n=32)	Group B (n=30)
Visit 1 (Day 0)	8 (2-16)^$$^	5 (1-15)
Visit 2 (Day 30 ± 3)	6 (1-15)*	4 (1-15)
Visit 3 (Day 60 ± 3)	3 (1-11)***^###^	3 (0-11)***^#^
Visit 4 (Day 90 ± 3)	2 (0-9)***^###^	2 (0-11)***^###^

**Figure 6:**
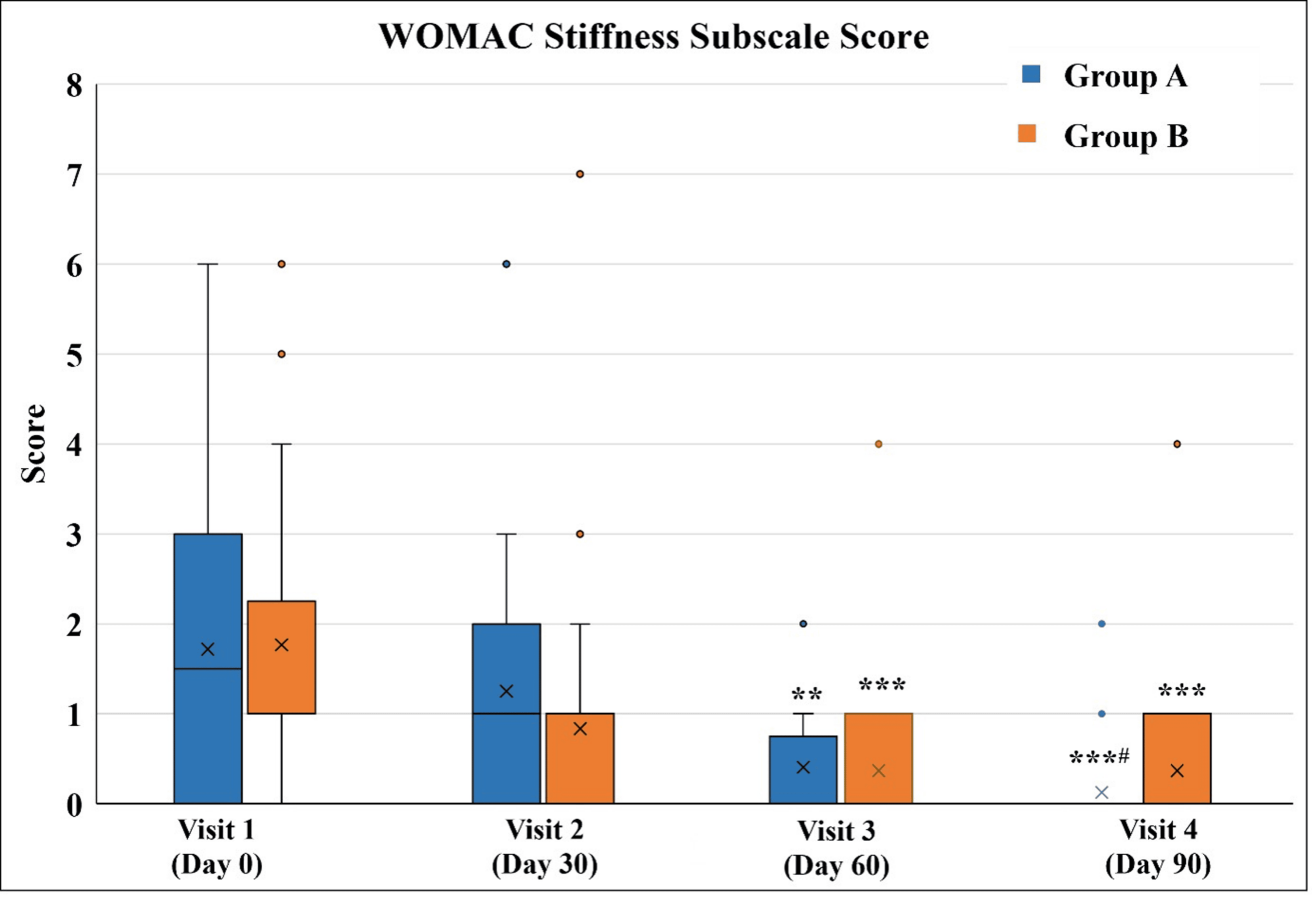
WOMAC Stiffness Subscale score ^**^p<0.01 and ^***^p<0.001 as compared to Visit 1; ^#^p<0.05 as compared to Visit 2 using the Friedman test. WOMAC: Western Ontario and McMaster Universities Osteoarthritis Index

In the case of the WOMAC Stiffness Subscale score, there was a significant decrease in both groups from Visit 3 (Figure 6 and Table 8).

**Table 8:** WOMAC Stiffness Subscale score ^*^p<0.05 and ^**^p<0.01, ^***^p<0.001 as compared to Visit 1; ^#^p<0.05 and ^###^p<0.001 as compared to Visit 2 using the Friedman test; ^$$^p<0.01 as compared to Group B using the Mann-Whitney test. WOMAC: Western Ontario and McMaster Universities Osteoarthritis Index

Visit	WOMAC Stiffness Subscale score	
	Group A (n=32)	Group B (n=30)
Visit 1 (Day 0)	1.5 (0-6)	1 (0-6)
Visit 2 (Day 30 ± 3)	1 (0-6)	0 (0-7)
Visit 3 (Day 60 ± 3)	0 (0-2)**	0 (0-4)***
Visit 4 (Day 90 ± 3)	0 (0-2)***^#^	0 (0-4)***

**Figure 7:**
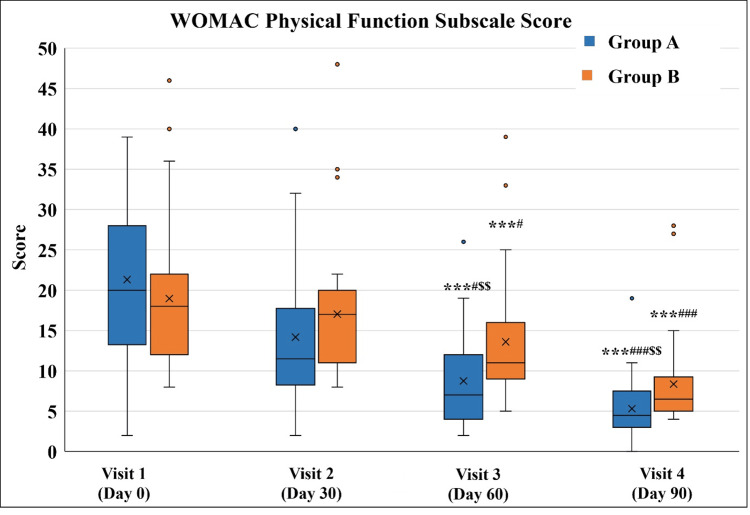
WOMAC Physical Function Subscale score ^***^p<0.001 as compared to Visit 1; ^#^p<0.05 and ^###^p<0.001 as compared to Visit 2 using the Friedman test; ^$$^p<0.01 as compared to Group B using the Mann-Whitney test. WOMAC: Western Ontario and McMaster Universities Osteoarthritis Index

A statistically significant decrease in WOMAC Physical Function Subscale Score was observed in both the groups at Visit 3 and Visit 4 as compared to Visit 1 and Visit 2. Group A exhibited a significantly lower score as compared to Group B on Visit 3 and Visit 4 (Figure 7 and Table 9).

**Table 9:** WOMAC Physical Function Subscale score ^***^p<0.001 as compared to Visit 1; ^#^p<0.05 and ^###^p<0.001 as compared to Visit 2 using the Friedman test; ^$$^p<0.01 as compared to Group B using the Mann-Whitney test. WOMAC: Western Ontario and McMaster Universities Osteoarthritis Index

Visit	Group A (n=32)	Group B (n=30)
Visit 1 (Day 0)	20 (2-39)	18 (8-46)
Visit 2 (Day 30 ± 3)	11.5 (2-40)	17 (8-48)
Visit 3 (Day 60 ± 3)	7 (2-26)***^#$$^	11 (5-39)***^#^
Visit 4 (Day 90 ± 3)	4.5 (0-19)***^###$$^	6.5 (4-28)***^###^

Comparison of the distance covered in the 6MWT in Group A and Group B 

**Figure 8:**
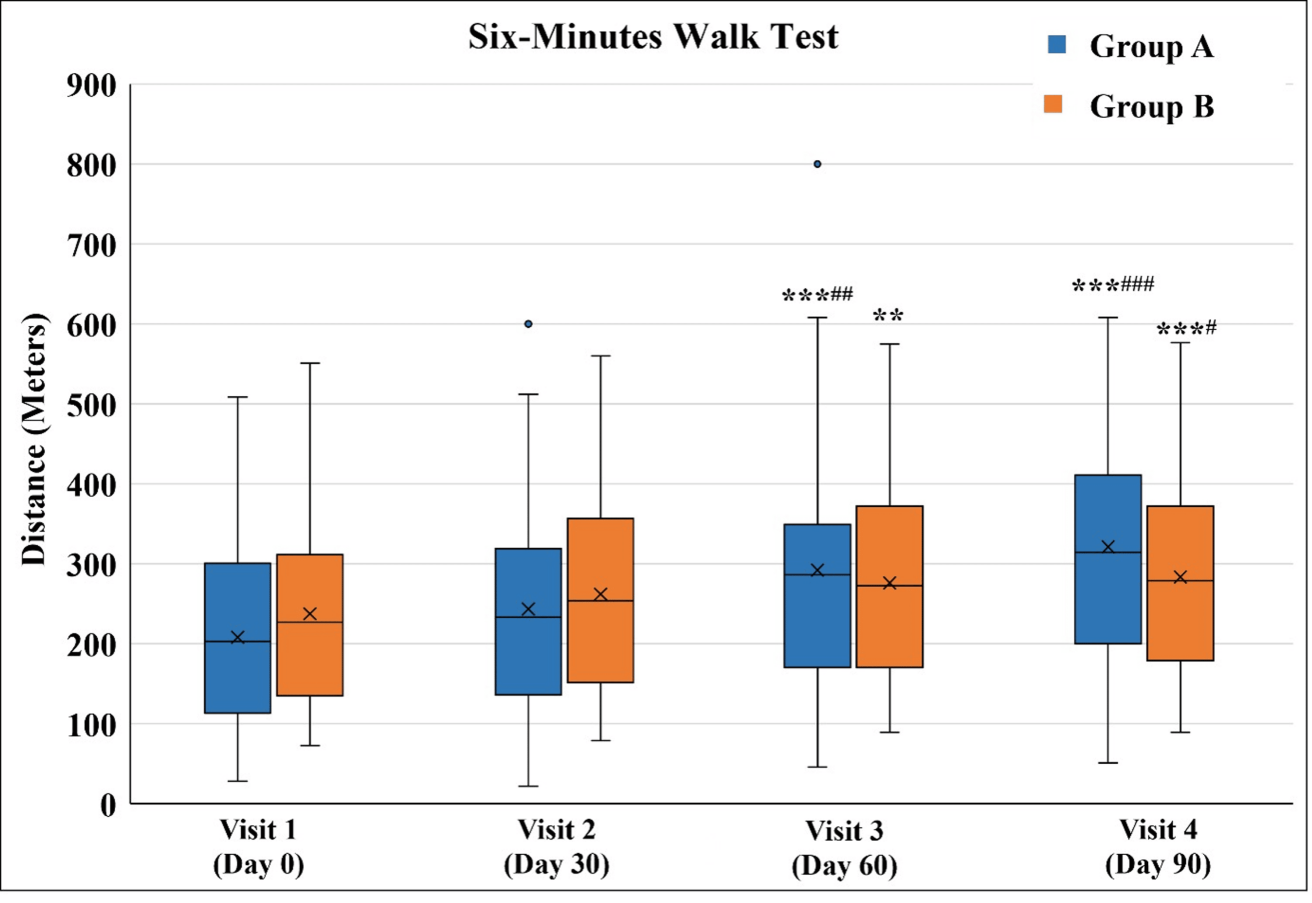
Six-Minute Walk Test ^**^p<0.01 and ^***^p<0.001 as compared to Visit 1; ^#^p<0.05, ^##^p<0.01, and ^###^p<0.001 as compared to Visit 2 using the Friedman test.

The distance covered in the 6MWT significantly improved in both groups from Visit 3 onwards as compared to the distance covered in Visit 1. Only in Group A, the distance covered on Visit 3 was significant as compared to Visit 2. Although there was no difference in both groups at any time point, the absolute change in the distance covered from Visit 1 to Visit 4 was significantly greater in Group A as compared to Group B (Figure 8 and Table 10).

**Table 10:** Six-Minute Walk Test ^**^p<0.01 and^***^p<0.001 as compared to Visit 1; ^#^p<0.05, ^##^p<0.01, and ^###^p<0.001 as compared to Visit 2 using the Friedman test; ^$$^p<0.01 as compared to Group B using the Mann-Whitney test.

Visit	Group A (n=32)	Group B (n=30)
Visit 1 (Day 0)	203. 25 (28.32-509)	203.5 (73-551)
Visit 2 (Day 30 ± 3)	233.5 (22-600)	206.5 (78.8-560)
Visit 3 (Day 60 ± 3)	286.5 (46-800)***^##^	231 (89-575)**
Visit 4 (Day 90 ± 3)	314.5 (51-845)***^###^	242.5 (89-577)***^#^
Absolute change from Visit 1 to Visit 4	85.96 (-42- 529)^$$^	28 (-41-191)

Comparison of change in the SLST duration in Group A and Group B

**Figure 9:**
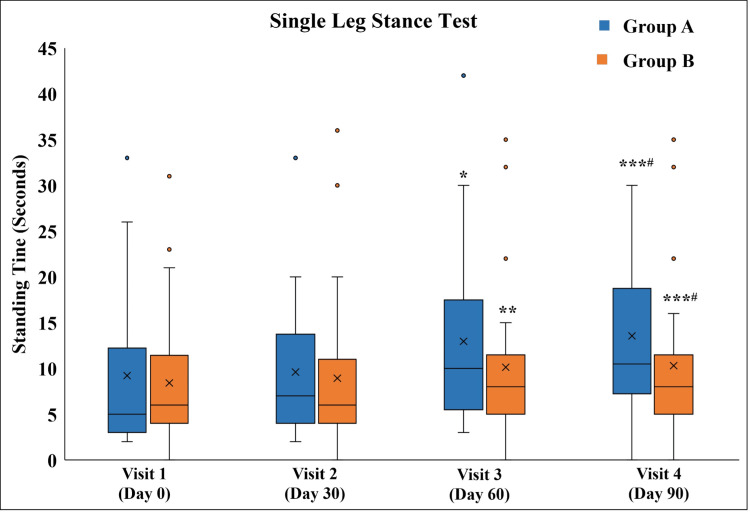
Single Leg Stance Test ^*^p<0.05, ^**^p<0.01, and ^***^p<0.001 as compared to Visit 1; ^#^p<0.05 as compared to Visit 2 using the Friedman test.

Both groups exhibited an increase in time for which the participants could stand on a single leg from Visit 3 onwards. The improvement on Visit 4 was statistically significant as compared to Visit 2. Although there was no difference in both groups at any time point, the absolute change in the increase in standing time from Visit 1 to Visit 4 was more significant in Group A as compared to Group B (Figure 9 and Table 11). In Group A, 10 participants were unable to stand on Visit 1. Of them, three were able to stand on Visit 3, and two were able to stand on Visit 4. In Group B, three participants were unable to stand on Visit 1, of which only one participant was able to stand on Visit 2.

**Table 11:** Single Leg Stance Test ^*^p<0.05, ^**^p<0.01, and ^***^p<0.001 as compared to Visit 1; ^#^p<0.05 as compared to Visit 2 using the Friedman test; ^$$$^p<0.01 as compared to Group B using the Mann-Whitney test.

Visit	Group A (n=22)	Group B (n=27)
Visit 1 (Day 0)	4 (2-33)	6 (3-31)
Visit 2 (Day 30 ± 3)	7 (2-33)	6.5 (1-36)
Visit 3 (Day 60 ± 3)	11 (3-33)*	8.5 (3-35)**
Visit 4 (Day 90 ± 3)	12 (5-30)***^#^	8.5 (4-35) ***^#^
Absolute change from Visit 1 to Visit 4	5 (-3 - 21) ^$$$^	2 (-8 - 12)

Comparison of change in the VAS score in Group A and Group B 

**Figure 10:**
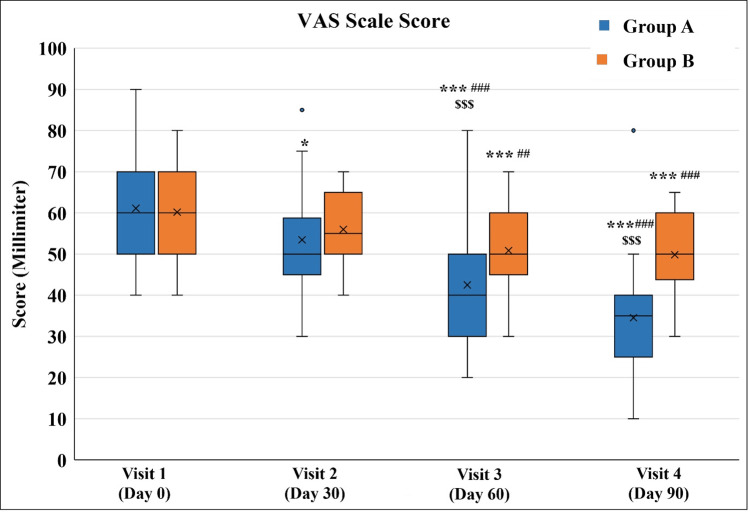
Change in the Visual Analogue Scale (VAS) score ^*^p<0.05 and ^***^p<0.001 as compared to Visit 1; ^##^p<0.01 and ^###^p<0.001 as compared to Visit 2 using the Friedman test; ^$$$^p<0.001 as compared to Group B using the Mann-Whitney test.

A significant decrease in VAS score was observed in both groups at Visit 3 and Visit 4 as compared to Visit 1. Group A exhibited a significant reduction from Visit 2, and a significant reduction in Group B from Visit 3 onwards. Group A exhibited a significantly lower VAS score as compared to Group B on Visit 3 and Visit 4. The decrease in VAS score seen in Group A on Visit 4 was almost 50% compared to Visit 1 (Figure 10 and Table 12).

**Table 12:** Change in the Visual Analogue Scale (VAS) score ^*^p<0.05, ^***^p<0.001 as compared to Visit 1 (Day 0), ^##^p<0.01, ^###^p<0.001 as compared to Visit 2 (Day 30 ± 3) using Friedman’s test. ^$$$^p<0.001 as compared to Group B using Mann Whitney test.

Visit	Group A (n=32)	Group B (n=30)
Visit 1 (Day 0)	60 (40-90)	60 (40-80)
Visit 2 (Day 30 ± 3)	50 (30-85)*	55 (40-70)
Visit 3 (Day 60 ± 3)	40 (20-80)***^###$$$^	50 (30-70)***^##^
Visit 4 (Day 90 ± 3)	35 (10-80)***^###$$$^	50 (30-65)***^###^

Number of participants showing adverse events in Group A as compared to Group B

The number of participants showing adverse events until Visit 4 was two out of 32 in Group A and six out of 30 in Group B. The adverse events observed in both groups were mild in the form of cough and cold and were not related to the study interventions. 

The participants in both groups, i.e., Group A and Group B, adhered well to the treatment. The compliance to Myostaal® liniment local application in the form of massage in Group A followed by the physiotherapy regimen and a physiotherapy regimen alone in Group B was in the acceptable range (≥80%).

Power analysis of the study

The calculated Cohen's d (effect size) for the difference in means at Visit 4 was 1.01, with a pooled standard deviation of 2.34, indicating a large effect size. Using this effect size and a significance level of α = 0.05, the study's power was calculated to be approximately 97.5%. This meant there was a very high probability (97.5%) of detecting a significant difference between the two groups if such a difference truly existed.

## Discussion

Osteoarthritis is a common chronic, non-communicable disease. The knee is the most frequently affected joint in OA that causes pain, reduces mobility, restricts activities of daily living, causes significant disability, and affects the quality of life [13]. The current standard management options for knee OA are weight loss, physiotherapy exercises, analgesics, intra-articular corticosteroid injections, and surgery in some advanced cases [14]. Many patients still fail to get relief from symptoms. Lower quadriceps strength is reported to contribute to poor subject-reported function, worse physical performance, and disease progression [15]. Further, it is reported that strengthening quadriceps muscle can also prove to be a good management strategy to improve pain, function, and quality of life of patients with knee OA [5]. Any physical interventions to strengthen the quadriceps at an appropriate stage will reduce the disease progression and prevent a worse functional outcome [15]. With the primary objective of knee muscle strengthening in cases of unilateral knee OA, the present study was planned to compare the efficacy of a physiotherapy regimen alone as compared to physiotherapy along with Myostaal® liniment local application (massage).

Sixty-two participants completed the study: 32 in Group A which was the test medication group, i.e., Myostaal® liniment local application as an add-on to a physiotherapy regimen, and 30 in Group B which was the standard treatment group, i.e., a physiotherapy regimen alone. It was observed that knee muscle strength (quadriceps muscle strength) was significantly higher in Group A as compared to Group B (9.80±2.74 vs. 7.43 ± 1.82) for the index knee after completion of the 90-day treatment period. 

Test medication and standard treatment were also advocated for the non-index knee. It was observed that Myostaal® liniment massage followed by a physiotherapy regimen significantly increased the knee muscle strength for the non-index knee after 90 days of treatment, although it was significantly low before the initiation of treatment. In brief, Myostaal® liniment application (massage) as an add-on therapy to a physiotherapy regimen significantly increased the quadriceps muscle strength in both index and non-index knees. This indicates its positive role in supporting knee joint health by providing symptomatic relief, improving functional efficiency, and also slowing down the progression of knee OA. 

The participants in both groups were also assessed on the WOMAC Index, which is a 24-item questionnaire focusing on pain, stiffness, and functional limitation. In resource-constrained settings, a simple questionnaire-based tool like the WOMAC Index is beneficial to assess the risk of knee OA and monitor the effectiveness of treatment [16].

In this study, the total WOMAC score and individual subscale scores of pain, stiffness, and physical function improved in both groups after 90 days of treatment. The improvement in Group A for total WOMAC score and Subscale score of Physical function was statistically more significant as compared to Group B, indicating a positive role of Myostaal® liniment application (massage) for better improvement in functional efficiency of the knee joint.

The VAS score (an indicator of pain) showed significant improvement in both groups after 90 days of treatment. This significance in Group A was seen from Day 30 onwards, and in Group B, it was seen from Day 60 onwards. There was a significant statistical difference between both groups after 60 and 90 days of treatment. These findings highlight the early and better pain-relieving effect of Myostaal® liniment.

Functional impairment resulting from knee OA can also be reliably evaluated with the 6MWT [17]. Both groups demonstrated significant improvement in 6MWT after 90 days of treatment as compared to their respective assessments before the initiation of treatment. However, the absolute change (difference) in distance traveled before treatment and after completion of treatment was statistically significant in Group A (median distance of 85.6 meters in Group A vs. 28 meters in Group B). This indicates better improvement in functional capacity after the application (massage) of Myostaal® liniment.

Knee OA is a major cause of disability and a risk factor for falls in older people, contributing to mobility limitations and difficulties with activities of daily living [18]. It is reported that older subjects with lower quadriceps muscle strength are at greater risk of falls [19]. The SLST is a reliable test to assess the risk of falling. Both groups exhibited an increase in time for which the participants could stand on a single leg after 90 days of treatment. However, the absolute change (difference) in the single leg standing time before treatment and after completion of 90 days of treatment was statistically significant in Group A (median time of five seconds in Group A vs. two seconds in Group B). This indicates better quadriceps muscle strength and knee stability after the application (massage) of Myostaal® liniment.

There were no major adverse effects reported during the study, thus assuring the safety of study interventions. Also, a higher number of participants in Group B reported the use of rescue medications as compared to Group A. The compliance/adherence to treatment in both groups was well within the acceptable range (≥80%).

The study's strengths include its randomized controlled design and is the first study to investigate Myostaal® liniment, which contributes to the robustness of the findings. The high statistical power of approximately 97.5% indicates that the study was well-equipped to detect significant differences within the sample. However, there are notable limitations. The sample size of 60 participants may restrict the generalizability of the results to the broader population with unilateral idiopathic knee OA. Additionally, the study was conducted at two sites within Parul University, which might introduce site-specific biases despite attempts at standardizing procedures. The dependence on self-reported adherence to the physiotherapy regimen could also introduce variability in the outcomes. A thorough analysis of these limitations, including potential biases and the implications for generalizability, is crucial for a comprehensive assessment of the study’s validity and applicability.

## Conclusions

The addition of Myostaal® liniment to a physiotherapy regimen demonstrated potential benefits in improving knee muscle strength and joint function in patients with knee OA. The local application of the liniment appeared to enhance pain relief and the functional efficiency of the joint. However, the study's limitations, such as the small sample size and site-specific factors, were important to consider as they might influence the generalizability of the results. These findings provided a basis for further research to confirm the effectiveness of Myostaal® liniment in diverse populations and to fully assess its role as an adjunct therapy in managing knee OA.

## References

[1] Sancheti P, Shetty VD, Dhillon MS, Sprague SA, Bhandari M (2017). India-based knee osteoarthritis evaluation (iKare): a multi-centre cross-sectional study on the management of knee pain and early osteoarthritis in India. Clin Orthop Surg.

[2] Vincent KR, Vincent HK (2012). Resistance exercise for knee osteoarthritis. PM R.

[3] Perlman AI, Sabina A, Williams AL, Njike VY, Katz DL (2006). Massage therapy for osteoarthritis of the knee: a randomized controlled trial. Arch Intern Med.

[4] Farr Ii J, Miller LE, Block JE (2013). Quality of life in patients with knee osteoarthritis: a commentary on nonsurgical and surgical treatments. Open Orthop J.

[5] Imoto AM, Peccin MS, Trevisani VF (2012). Quadriceps strengthening exercises are effective in improving pain, function and quality of life in patients with osteoarthritis of the knee. Acta Ortop Bras.

[6] Jain A, Panchal Y, Choursiya D, Adlak B, Khan AU (2023). Advanced automation of janu dhara and janu basti process in Ayurveda. Int J Health Tech Innovat.

[7] Das B, Padhi MM, Singh OP, Deep VC, Tewari NS, Panda N (2003). Clinical evaluation of nirgundi taila in the management of sandhivata. Anc Sci Life.

[8] Uriah T, Rai S, Mohanty JP, Ghosh P (2019). Physicochemical evaluation, in vitro anti-inflammatory, in vitro anti-arthritic activities and GC-MS analysis of the oil from the leaves of Gaultheria fragrantissima Wall of Meghalaya. Journal of Drug Delivery and Therapeutics.

[9] Chandorkar N, Tambe S, Amin P, Madankar C (2021). A systematic and comprehensive review on current understanding of the pharmacological actions, molecular mechanisms, and clinical implications of the genus Eucalyptus. Phytomed Plus.

[10] Bisht A, Jain S, Misra A, Dwivedi J, Paliwal S, Sharma S (2021). Cedrus deodara (Roxb. ex D.Don) G.Don: a review of traditional use, phytochemical composition and pharmacology. J Ethnopharmacol.

[11] Labib RM, Youssef FS, Ashour ML, Abdel-Daim MM, Ross SA (2017). Chemical composition of Pinus roxburghii bark volatile oil and validation of its anti-inflammatory activity using molecular modelling and bleomycin-induced inflammation in albino mice. Molecules.

[12] Askari A, Ravansalar SA, Naghizadeh MM, Mosavat SH, Khodadoost M, Jazani AM, Hashempur MH (2019). The efficacy of topical sesame oil in patients with knee osteoarthritis: a randomized double-blinded active-controlled non-inferiority clinical trial. Complement Ther Med.

[13] Bijlsma JW, Berenbaum F, Lafeber FP (2011). Osteoarthritis: an update with relevance for clinical practice. Lancet.

[14] Liow Y, Wang W, Loh VW (2017). Outpatient management of knee osteoarthritis. Singapore Med J.

[15] Hegde S, Ranganath N (2021). Assessment of quadriceps muscle weakness in association with symptomatic and radiological osteoarthritis of the knee. medRxiv.

[16] Sathiyanarayanan S, Shankar S, Padmini SK (2017). Usefulness of WOMAC index as a screening tool for knee osteoarthritis among patients attending a rural health care center in Tamil Nadu. Int J Community Med Public Health.

[17] Matos Casano HA, Anjum F (2024). Six-Minute Walk Test. https://www.ncbi.nlm.nih.gov/books/NBK576420/.

[18] Hatfield GL, Morrison A, Wenman M, Hammond CA, Hunt MA (2016). Clinical tests of standing balance in the knee osteoarthritis population: systematic review and meta-analysis. Phys Ther.

[19] Ahmadiahangar A, Javadian Y, Babaei M, Heidari B, Hosseini S, Aminzadeh M (2018). The role of quadriceps muscle strength in the development of falls in the elderly people, a cross-sectional study. Chiropr Man Therap.

